# Early prediction of infarct size by quantitative myocardial blush grade in patients with acute non-st-elevation and ST-elevation myocardial infarction treated with primary angioplasty and stent placement

**DOI:** 10.1186/1532-429X-11-S1-P186

**Published:** 2009-01-28

**Authors:** Nina Riedle, Hartmut Dickhaus, Markus Erbacher, Henning Steen, Martin Andrassy, Dirk Lossnitzer, Stefan Hardt, Wolfgang Rottbauer, Christian Zugck, Hugo A Katus, Evangelos Giannitsis, Grigorios Korosoglou

**Affiliations:** 1 Department of Cardiology, University of Heidelberg, Heidelberg, Germany; 2 Department of Medical Informatics, University of Heidelberg, Heidelberg, Germany

**Keywords:** Infarct Size, Stent Placement, Microvascular Obstruction, Primary Angioplasty, Artery Blood Flow

## Introduction

The widespread use of percutaneous coronary interventions has resulted in a significant improvement of clinical outcomes in patients both with ST-elevation and in non-ST-elevation myocardial infarction (STEMI and NSTEMI). The restoration of epicardial artery blood flow in the revascularized coronary artery however, may not necessarily guarantee preserved microvascular integrity in the downstream myocardium. Because the latter is a principal predictor of clinical outcomes, methods that can provide objective assessment of infarct size early in acute infarction may be of great potential clinical utility.

## Purpose

To determine whether quantification of myocardial blush grade (MBG) can aid the determination of infarct size during cardiac catheterization in patients with acute myocardial infarction.

## Methods

We prospectively examined patients with first STEMI (n = 45) and NSTEMI (n = 50), all treated with primary angioplasty and stent placement. ECG-gated angiographic series were used to quantify MBG, by placing regions of interest (ROI) in the infarct related coronary territory, in order to estimate the time course of blush intensity rise. G_max_ was defined as the peak grey level intensity and T_max_ as the time to peak intensity rise. By this approach, we anticipated that an adequate and prompt filling of myocardial capillaries with contrast agent (high G_max_ within a short T_max_) would be indicative of preserved microvascular integrity and predictive of small infarct size and vice versa. Assessment of myocardial scar, determined by contrast-enhanced magnetic resonance imaging (MRI), 2 to 4 days after infarction, deemed as the standard reference for estimation of infarct size. Briefly, 10 minutes after 0.2 mmol/kg body weight gadolinium contrast injection, 2 volume stacks of inversion-recovery gradient-echo images covering the whole left ventricle were generated. Infarct size was calculated as the amount of hyperenhanced myocardium related to the total LV-mass. Infarct transmurality was assessed visually based on a 5-grade scale (i.e. 1 = 0–25%, 2 = 25–50%, 3 = 50–75%, 4/5 = 75–100% transmurality without/with microvascular obstruction).

## Results

**Figure 1:**
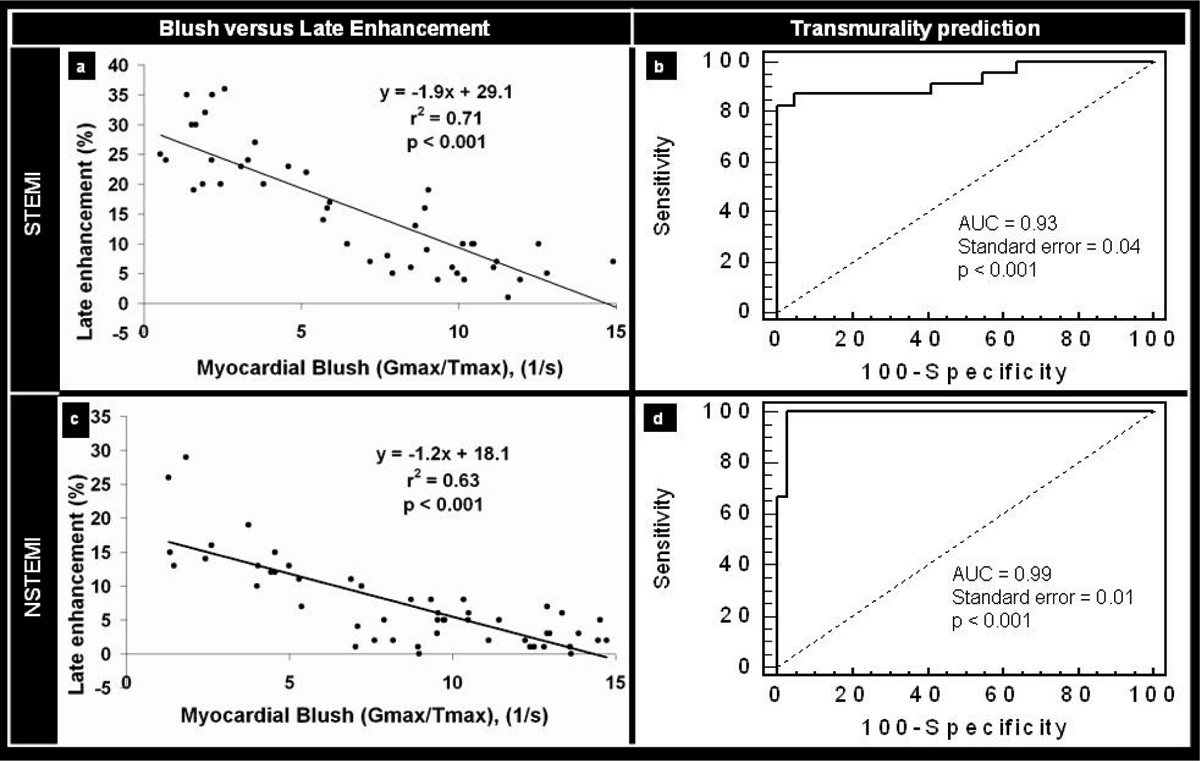
Figure 1

**Figure 2:**
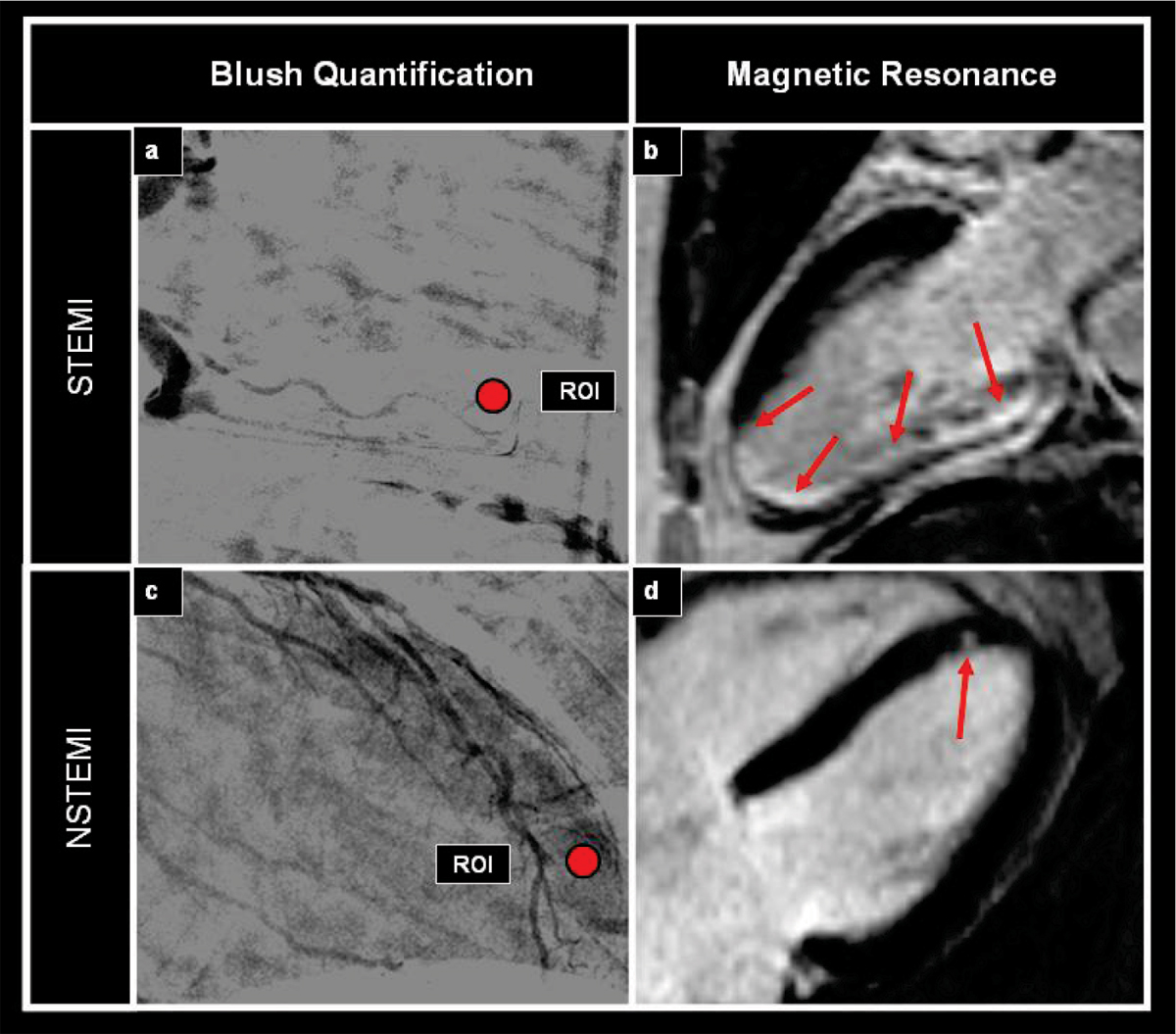
Figure 2

Patients with STEMI showed larger infarct size compared to patients with NSTEMI, both by cardiac enzymes (peak troponin T of 7.3 ± 6.8 μg/l for STEMI vs. 2.3 ± 2.5 μg/l for NSTEMI, p < 0.01) and by delayed enhancement on MR-images (15.7 ± 9.9% for STEMI vs. 7.5 ± 6.7% for NSTEMI, p < 0.01). The ratio G_max_/T_max_ showed a significant inverse linear correlation with infarct size both in patients with STEMI and in those with NSTEMI (r^2^ = 0.71 vs. r^2^ = 0.63, p < 0.001), (Figure 1a,c). Furthermore, G_max_/T_max_ was significantly related with infarct transmurality (χ^2^ = 42.7 for STEMI and χ^2^ = 51.7 for NSTEMI, respectively, p < 0.001) and cut-off values of G_max_/T_max_ = 5.9/s and 5.0/s, respectively were highly predictive for the presence of infarct transmurality = 75% in both groups (AUC = 0.93, 95%CI = 0.81–0.98 for STEMI and AUC = 0.99, 95%CI = 0.91–0.99 for NSTEMI, respectively, p < 0.001), (Figure 1b,d). MR- and angiographic findings of a large transmural inferior wall STEMI with reduced MBG (Figure 2a,b) and of a small subendocardial anterior wall NSTEMI (Figure 2c,d) with higher MBG can be appreciated in corresponding figures.

## Conclusion

Quantitative MBG is a valuable predictor of the total extent of myocardial infarction and infarct transmurality. This information can be easily acquired during clinically indicated cardiac catheterization, immediately after myocardial reperfusion, and can be utilized for tailoring appropriate pharmacological interventions and to support the early risk stratification of patients with acute ischemic syndromes.

